# Supplementary material for: Enhanced Power Generation by Piezoelectric P(VDF-TrFE)/rGO Nanocomposite Thin Film
Source: Nanomaterials (Basel). 2023 Feb 25;13(5):860. doi: 10.3390/nano13050860 (PMC10005479; doi:10.3390/nano13050860)
Supplement: Supplementary file 1 [file nanomaterials-13-00860-s001.zip › nanomaterials-2242121-supplementary.pdf]

Supplementary Materials

Enhanced Power Generation by Piezoelectric P(VDF-TrFE)/rGO Nanocomposite Thin Film

Hafiz Muhammad Abid Yaseen, Sangkwon Park*

Department of Chemical and Biochemical Engineering, Dongguk University, 30, Pildong-Ro 1 Gil, Jung-Gu, Seoul, 04620, South Korea

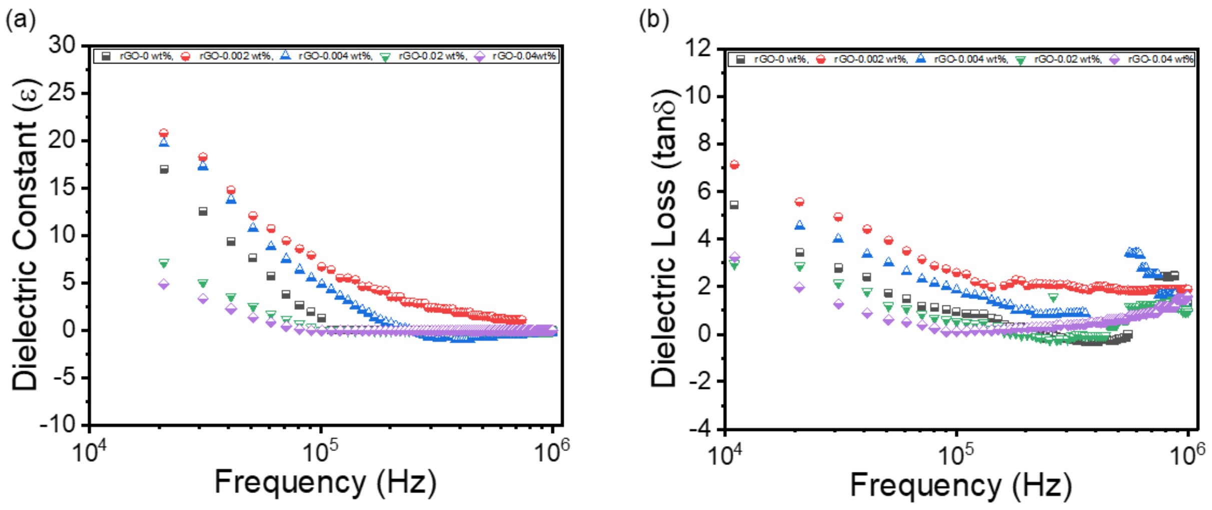

Figure S1. (a) Dielectric constant (ε); (b) Dielectric loss (tan δ) of five thin films.

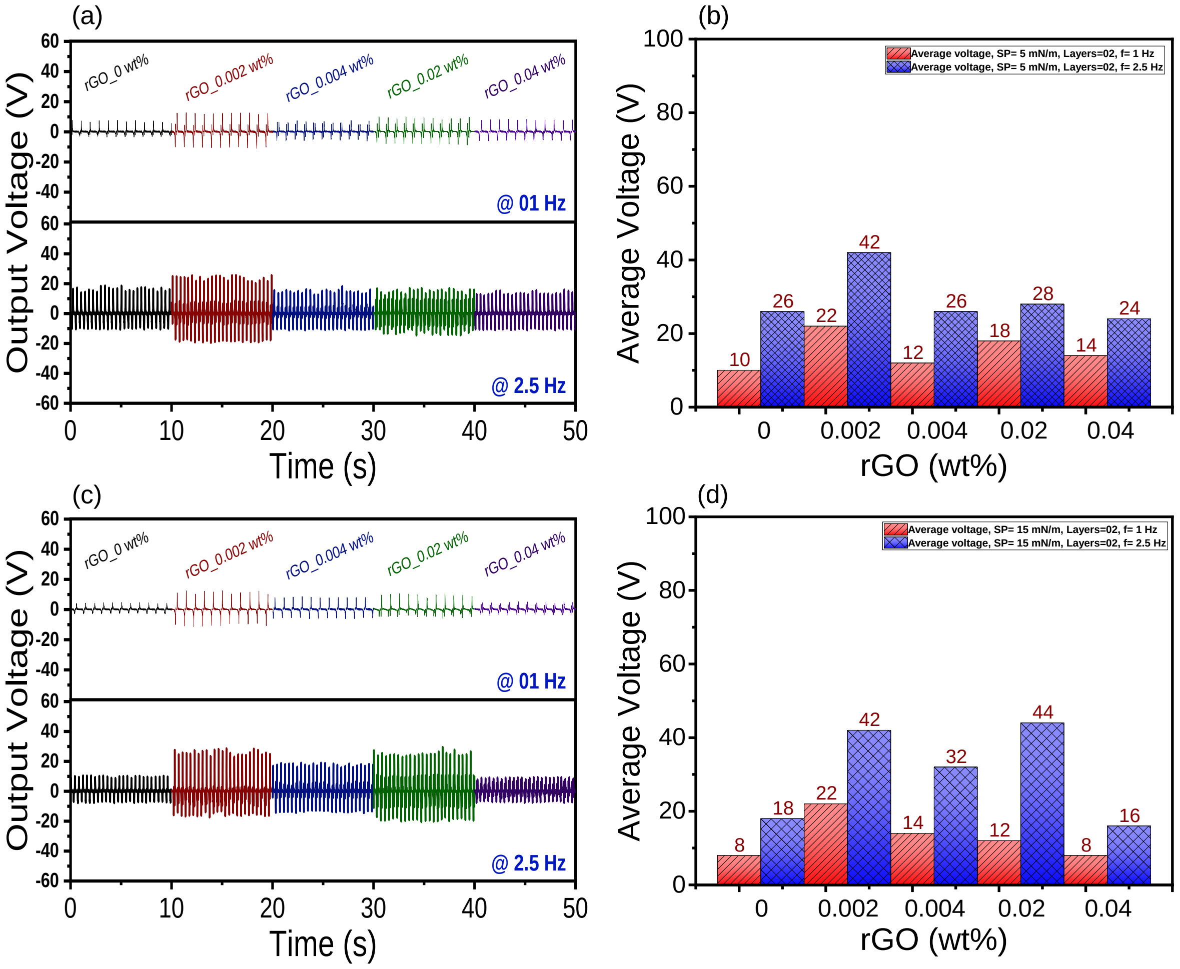

Figure S2. (a) V_{OC} signals generated by five PENGs with two monolayers at π = 5 mN/m; (b) average peak-peak V_{OC} of five PENGs at π = 5 mN/m; (c) V_{OC} signals generated by five PENGs with two monolayers at π = 15 mN/m; (d) average peak-peak V_{OC} of five PENGs at π = 15 mN/m.

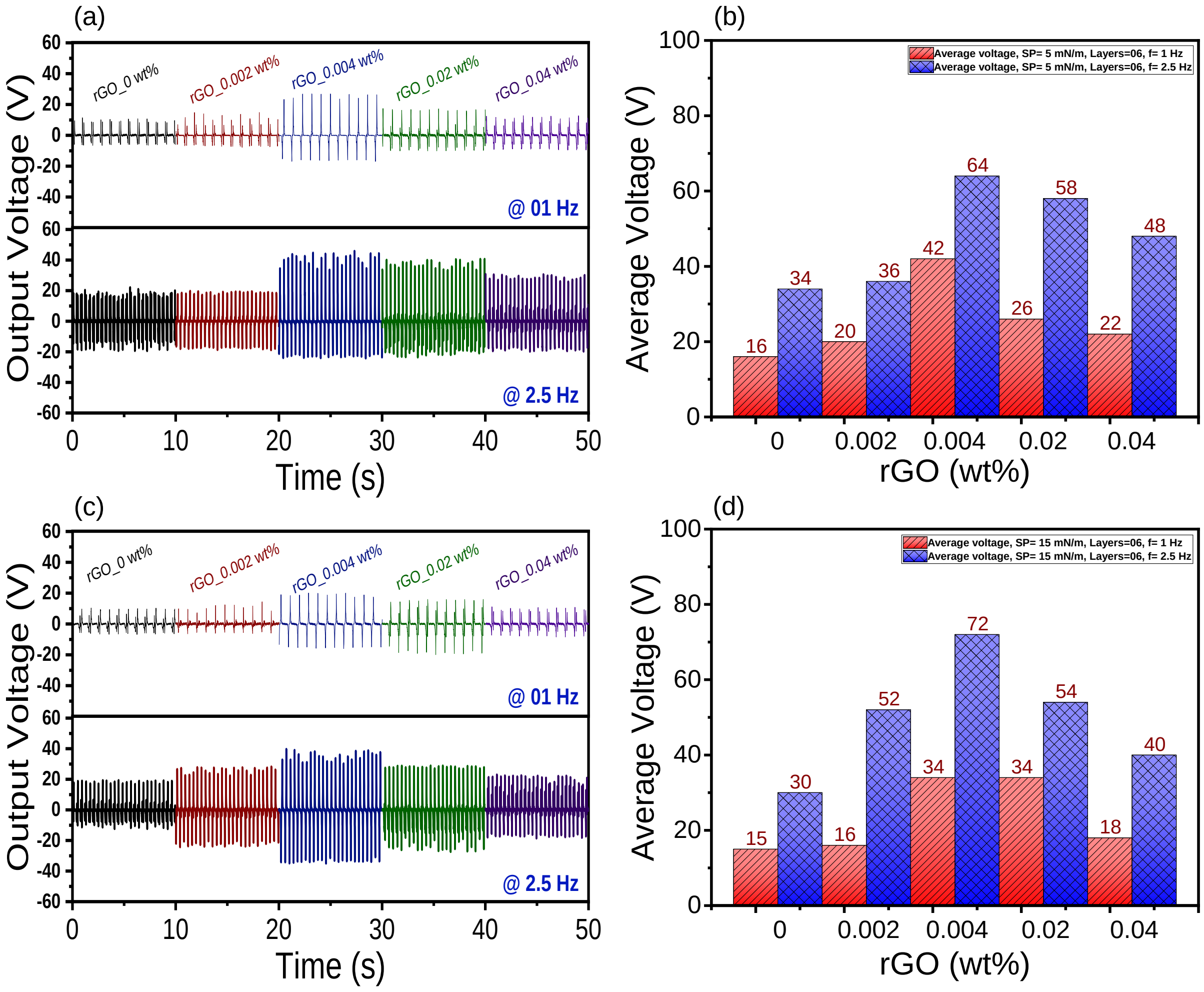

Figure S3. (a) V_{OC} signals generated by five PENGs with six monolayers at π = 5 mN/m; (b) average peak-peak V_{OC} of five PENGs at π = 5 mN/m; (c) V_{OC} signals generated by five PENGs with six monolayers at π = 15 mN/m; (d) average peak-peak V_{OC} of five PENGs at π = 15 mN/m.

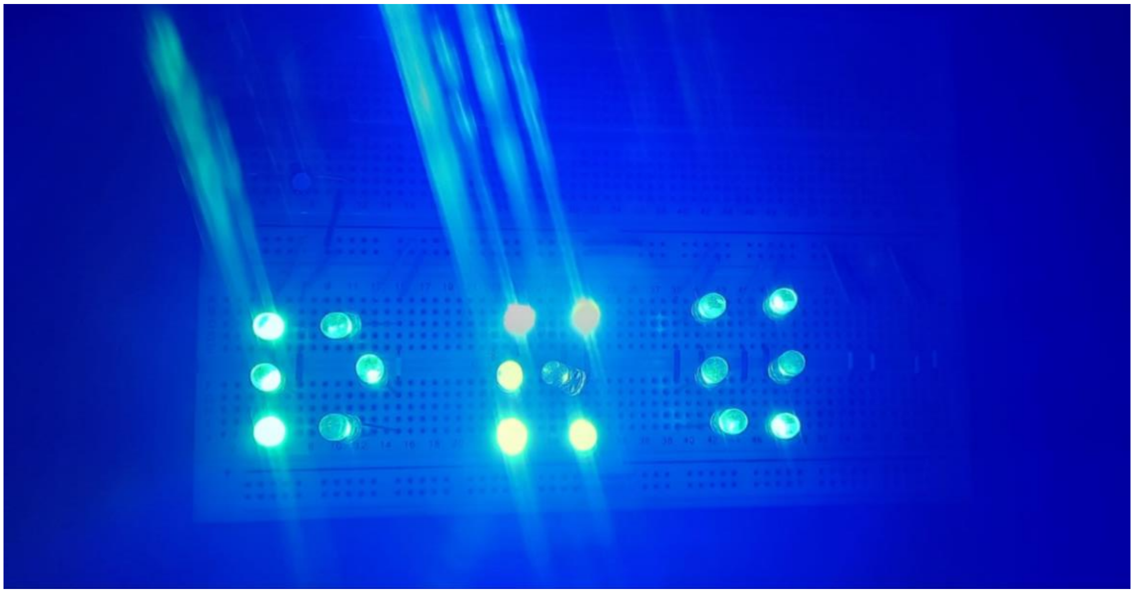

Figure S4. Glowing LEDs operated by the circuit in Figure 8a.
